# Jolkinolide B Mitigates Cerebral Ischemia–Reperfusion Injury by Promoting Microglial M1/M2 Polarization Through the JAK2/STAT3 Signaling Pathway

**DOI:** 10.1111/cns.70653

**Published:** 2025-11-18

**Authors:** Yupeng Guo, Xuanwei Dong, Min Liu, Dongsheng Liu, Jianxin Wang, Shewei Guo

**Affiliations:** ^1^ Department of Neurosurgery Aviation General Hospital Beijing China; ^2^ Neurosurgical Department The First Affiliated Hospital of Zhengzhou University Zhengzhou Henan China

**Keywords:** CIRI, ischemic stroke, JAK2/STAT3, Jolkinolide B, microglial polarization

## Abstract

**Background:**

Microglia polarization and inflammatory response are closely related to cerebral ischemia–reperfusion injury (CIRI). The diterpenoid compound Jolkinolide B (JB) possesses anti‐inflammatory properties, but the effects of JB and the mechanism on CIRI remain unclear.

**Methods:**

An middle cerebral artery occlusion/reperfusion (MCAO/R) rat model and an oxygen–glucose deprivation/reoxygenation (OGD/R)‐induced HAPI cell model were used to evaluate the neuroprotective effects and mechanisms of JB. Neurological deficits and histopathological changes were assessed using Longa scoring, corner turn tests, TTC, HE, Nissl, and TUNEL staining. ELISA, flow cytometry, Western blot, and immunofluorescence were employed to analyze pro‐inflammatory cytokines, JAK2/STAT3 pathway proteins, and microglial polarization. In vitro, JB's effects on cell viability and apoptosis were evaluated using CCK‐8 and LDH release assays. Validation experiments were conducted using the JAK2‐specific inhibitor WP1066 and activator Broussonin E.

**Results:**

JB exhibited dose‐dependent neuroprotective effects in MCAO/R rats, improving neurological function, reducing infarction area, neuronal apoptosis, cerebral edema, and neuroinflammation. JB suppressed JAK2/STAT3 signaling by downregulating p‐JAK2, p‐STAT3, and M1 markers (iNOS, CD16) while upregulating M2 markers (Arg‐1, CD206) and reducing pro‐inflammatory cytokines (IL‐1β, TNF‐α, IFN‐γ). Both in vivo and in vitro, JB inhibited the JAK2/STAT3 signaling pathway and promoted microglial polarization from M1 to M2, alleviating CIRI. In vitro, JB enhanced HAPI cell viability, decreased apoptosis, and reduced LDH leakage.

**Conclusion:**

The ability of JB to modulate microglial polarization through JAK2/STAT3 inhibition presents a promising pharmacological approach for cerebral ischemia–reperfusion injury management in stroke therapy.

## Graphical abstract

Jolkinolide B improves ischemia–reperfusion injury by inhibiting the JAK2/STAT3 signaling pathway, regulating the polarization of microglia from the M1 to the M2 phenotype, and suppressing the release of pro‐inflammatory cytokines.

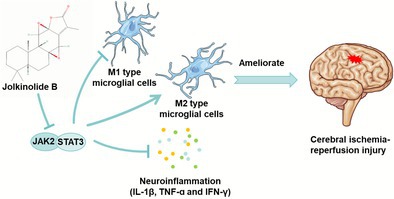

**Abbreviations**
CIA: collagen‐induced arthritis
CIRI: cerebral ischemic reperfusion injury
CUMS: chronic unpredictable mild stress
ECA: external carotid artery
ELISA: enzyme‐linked immunosorbent assay
HE: hematoxylin–eosin
ICA: internal carotid artery
JAK: Janus kinases
JB: Jolkinolide B
LDH: lactate dehydrogenase
MAPK: mitogen‐activated protein kinase
MCAO/R: middle cerebral artery occlusion/reperfusion
OGD/R: oxygen–glucose deprivation/reperfusion
PKC: protein kinase C
PVDF: polyvinylidene fluoride
RA: rheumatoid arthritis
STAT: signal transducer and activator of transcription
TTC: 2,3,5‐triphenyltetrazolium chloride
vmPFC: ventromedial prefrontal cortex

## 1 Introduction

Among neurological disorders, ischemic stroke (IS) is responsible for substantial global disability and fatal consequences [1, 2]. Currently, blood flow restoration in ischemic brain regions is primarily achieved through thrombolytic therapy in IS treatment. However, the therapeutic window for thrombolysis is limited, and reperfusion following ischemia may lead to further tissue injury, scientifically characterized as cerebral ischemia reperfusion injury (CIRI) [3, 4]. CIRI exacerbates neuronal cell death, triggers more extensive brain tissue dysfunction, thereby worsening brain injury and increasing rates of disability and mortality [5]. Elucidating CIRI's molecular pathways and identifying viable drug targets holds considerable clinical value for optimizing IS management.

Neuroinflammation plays a pivotal role in CIRI pathogenesis [6]. Microglia, serving as the CNS's resident immune cells, are essential regulators of neural immune balance [7]. After reperfusion, activated M1‐polarized microglia initiate the secretion of proinflammatory mediators such as TNF‐α, IL‐6, and IL‐1β [8], which induce a robust inflammatory response that exacerbates cerebral ischemic injury; whereas M2‐polarized microglia can release anti‐inflammatory factors like Arg‐1, TGF‐β, and IL‐4, which alleviate local inflammation, promote neurogenesis, and facilitate tissue repair [9, 10]. Therefore, modulating microglial polarization states represents a promising strategy to mitigate CIRI.

Jolkinolide B (JB), an aromatic abietane diterpenoid first characterized from Euphorbia jolkini [11], represents a pharmacologically significant compound found in multiple toxic Euphorbia species. This bioactive molecule exhibits diverse therapeutic properties spanning antineoplastic, anti‐inflammatory, bone‐protective, and antimicrobial activities [12, 13]. And JB exerts its pharmacological effects by suppressing critical cascades, including the Janus kinases/signal transducer and activator of transcription (JAK/STAT) axis, NF‐κB‐mediated inflammation, and the PI3K/Akt/mTOR pathway [14, 15, 16]. The JAK2/STAT3 pathway serves as a master regulator of microglial activation states and functional phenotypes. Suppressing the JAK2/STAT3 signaling pathway can decrease M1 microglial activation while facilitating M2 microglial polarization, thus achieving anti‐inflammatory and tissue‐repairing effects. This mechanism has been validated in diseases such as periodontitis, myocardial ischemia/reperfusion injury, and ulcerative colitis [17, 18, 19]. In studies of CIRI, activation of the JAK2/STAT3 pathway can promote the polarization of microglia toward the M2 phenotype [20]. Moreover, studies have found that in rheumatoid arthritis (RA), JB alleviates the progression of arthritis in collagen‐induced arthritis (CIA) rats by inhibiting this signaling pathway [14]. Nevertheless, the impact of JB on CIRI and its precise modulatory effects on microglial polarization states and associated inflammatory signaling pathways have yet to be fully elucidated. This study aims to elucidate JB's modulation of microglial M1/M2 polarization through JAK2/STAT3 pathway inhibition, evaluating its therapeutic potential for CIRI. These findings may offer innovative mechanistic perspectives and candidate drug targets for ischemic stroke management.

## 2 Materials and Methods

### 2.1 Animal

One hundred and eight young adult male Sprague–Dawley rats (250–300 g) were maintained in climate‐controlled conditions (20°C–22°C, 50%–55% humidity) with 12 h photoperiods. After one week of acclimation with free access to food and water in sanitized housing, all experiments were performed per the ethical standards approved by The First Affiliated Hospital of Zhengzhou University Animal Ethics Committee (Approval No.: 2021090201). Animals were sourced from Anhui Medical University's certified facility (Hefei, China).

### 2.2 Animal Grouping and Treatment

The CIRI model was established using the middle cerebral artery occlusion/reperfusion (MCAO/R) method [21]. Following overnight fasting, rats were anesthetized (2% sodium pentobarbital solution, injection volume of 50 μL) until no response was observed in the tail‐flick test. To induce focal cerebral ischemia, the right common carotid artery, external carotid artery (ECA), and internal carotid artery (ICA) were carefully exposed. The ECA was permanently tied off at its distal end. A 4–0 nylon monofilament with a rounded tip was then introduced through the ECA lumen and pushed forward about (18 ± 2) mm to block the middle cerebral artery's blood supply. This vascular blockade lasted for 90 min before the filament was withdrawn to allow reperfusion. Control animals received identical surgical treatment, but the filament was advanced only 5 mm without causing vascular occlusion. Throughout the operative procedure, core body temperature was regulated at 37°C ± 0.5°C using a feedback‐controlled heating system.

The 108 rats were randomly assigned to 6 groups (*n* = 12): (1) Sham group; (2) MCAO/R group; (3) MCAO/R + 7.5 mg/kg JB group; (4) MCAO/R + 15 mg/kg JB group; (5) MCAO/R + 30 mg/kg JB group; (6) MCAO/R + 30 mg/kg JB + 0.3 mg/kg Broussonin E (BE, JAK2 activator, 90902‐21‐9, BioBioPha). Beginning at the start of reperfusion, JB (216788470361377, HuicH) was administered intraperitoneally at a dose of once daily for 3 consecutive days [22]. The drugs were dissolved in DMSO to prepare stock solutions, which were then mixed with normal saline to prepare working solutions. The working solutions were administered by intraperitoneal injection at a volume of 5 μL/g body weight (equivalent to 5 mL/kg). An equivalent volume of 0.9% saline, adjusted according to body weight, was administered to rats in both the MCAO/R and sham‐operated groups.

### 2.3 Neurobehavioral Assessment

To quantify the extent of neurological functional impairment in each group of rats (*n* = 18), Longa scoring and the Corner test were conducted 3 days after MCAO/R. The Longa scoring criteria range from 0 points (no neurological deficit) to 4 points (unable to walk spontaneously, loss of consciousness) (Table 1). Rats with neurological deficits tend to use their unaffected left limbs for turning in the Corner test, resulting in an increased number of right turns. Rats were placed within a 30° angle formed by two boards (30 × 20 × 1 cm) to induce turning. Each subject completed ten test trials (≥ 1 min apart), and right turns were tallied for individual animals.

**TABLE 1:** Longa scoring criteria range.

Performance	Score
No neurological deficit, normal animal behavior	0
Mild neurological deficit, the animal cannot fully extend the contralateral forelimb	1
Moderate neurological deficit, the animal circles to the contralateral side	2
Severe neurological deficit, the animal leans toward the contralateral side	3
Incapable of spontaneous walking, loss of consciousness	4

### 2.4 Brain Water Fraction Assessment

The wet‐dry weight method was employed to quantify brain tissue hydration levels [23]. Brain tissues from 6rats per group were collected, and residual blood was washed off using ice‐chilled PBS. Excess liquid was removed with absorbent paper, and the tissues were immediately weighed on a petri dish to determine the wet weight. Brain tissue was dried in a forced air oven at 100°C for 36 h to measure its dry weight. Brain water content (%) = (wet weight − dry weight)/wet weight × 100%.

### 2.5 2,3,5‐Triphenyltetrazolium Chloride (TTC) Staining

Subsequent to behavioral analysis, a random selection of 6rats per treatment group was anesthetized for terminal euthanasia. Subsequently, the whole brains were frozen for 15 min at −20°C, then immediately sliced into 2 mm coronal sections which were immersed in 2% TTC solution (17779, Sigma‐Aldrich) at 37°C for 30 min, flipped every 5 min, and then the staining solution was discarded. The sections were fixed in 4% paraformaldehyde (PFA) for 30 min, laid flat, photographed using a digital camera (A7 IV, SONY), and analyzed with Image J software for calculation.

### 2.6 Hematoxylin–Eosin (HE) Staining and Nissl Staining

Histopathological evaluation was performed using HE and Nissl staining protocols. Right cerebral hemispheres were fixed in 4% PFA (4°C, 24 h), processed through graded ethanol dehydration, paraffin‐embedded, and sectioned at 4 μm thickness. After dewaxing and hydration, sections were stained with hematoxylin (2 min, G1120, Solarbio), rinsed (10 min), differentiated in 1% acid alcohol (3 min), counterstained with eosin (1 min), dehydrated, and then coverslipped with neutral balsam (G8590, Solarbio). Cortical tissue architecture was examined microscopically (BX53, Nikon), with images captured for analysis. Nissl staining was conducted according to the instructions of the Nissl Staining Kit (G1434, Solarbio), with dewaxed and hydrated sections stained in cresyl violet solution at 37°C for 1 h, processed in Nissl differentiation solution for 3 min to visualize Nissl bodies clearly, followed by a 3‐min treatment with ammonium molybdate solution, rinsing, and mounting with coverslips. Five microscopic fields (non‐repeating) were randomly identified for analysis. Nissl bodies in each area were counted using Image J software to assess brain damage.

### 2.7 Cell Culture and Oxygen–Glucose Deprivation and Reperfusion (OGD/R) Model

Primary rat microglial cells (HAPI cells) were purchased from Shanghai Enzyme Research Biotechnology Co. Ltd. (Shanghai, China). The cells were cultured for two weeks under stable conditions in a 37°C incubator with 5% CO_2_, using DMEM medium (11965092, Invitrogen) supplemented with 10% FBS (A5669701, Invitrogen), 100 U/mL penicillin, and 100 μg/mL streptomycin (C0222, Beyotime), with the medium changed every three days. When the cells reached confluence and a density of 90%, they were passaged. HAPI cells between passages 15 and 25 were used for subsequent experiments. The OGD/R method was used to treat HAPI cells, thereby simulating CIRI in vitro [24].

Cells were seeded into culture plates at a density of 5 × 10^4^ cells/cm^2^. During the establishment of the OGD/R model, cells were washed with PBS and incubated in glucose‐depleted DMEM medium (11966025, Invitrogen). Subsequently, the cells were exposed to hypoxia (95% N_2_ and 5% CO_2_) at 37°C for 2 h, followed by 24 h of normoxic culture in standard glucose‐containing DMEM medium.

### 2.8 Cell Treatment

After incubation with different concentrations of JB (0, 2.5, 5, 10, 20, 40 μM) for 24 h, HAPI cells were subjected to OGD/R treatment. Cell viability was measured via CCK‐8 assay to establish the working concentration range.

To investigate the mechanism of the JAK2/STAT3 signaling pathway in microglial polarization, this study employed a JAK2 activator (BE) and a JAK2 inhibitor (WP1066) to pretreat microglial cells. The experiment was divided into three groups: control group, 20 μM BE group, and 10 μM WP1066 group, to verify the effects of BE and WP1066 on the JAK2/STAT3 pathway.

Subsequently, 5 experimental groups were established: (1) Control group, with no treatment; (2) OGD/R group; (3) OGD/R + 10 μM JB group, where HAPI cells were incubated with 10 μM JB for 24 h; (4) OGD/R + 10 μM WP1066 group: Cells were pretreated with 10 μM WP1066 (SD4749, Beyotime) for 6 h before OGD/R; (5) OGD/R + 10 μM JB + 20 μM BE group: Cells were treated with 10 μM JB and 20 μM BE (90902‐21‐9, BioBioPha) for 3 h, followed by 24 h of incubation after OGD/R treatment. All drugs were dissolved in DMSO to prepare working solutions, and the control group received an equivalent amount of DMSO.

### 2.9 Cell Viability Assay

HAPI cells were plated in 96‐well plates (5 × 10^3^ cells/well) and cultured in 100 μL complete medium supplemented with varying concentrations of JB (0, 2.5, 5, 10, 20, 40 μM). Following 24 h culture, 10 μL CCK‐8 solution (A311‐01, Vazyme) was introduced to each well and maintained at 37°C in an incubator (CCL‐170T‐8, ESCO) for 3 h. Absorbance measurements were performed at 450 nm using a microplate reader (Multiskan FC, Thermo Fisher). Cell viability (%) was calculated as follows: [(OD_treatment_ − OD_blank_)/(OD_control_ − OD_blank_)] × 100%.

To investigate the combined effects of different concentrations of JB and OGD/R treatment on HAPI cell viability, the experimental procedure was the same. Specifically, prior to OGD/R treatment, HAPI cells were pre‐incubated with JB at the aforementioned concentrations for 24 h. Cells were then subjected to OGD/R following standard procedures, with viability assessed by CCK‐8 analysis.

### 2.10 TUNEL Staining

The apoptosis of neuronal cells in rat brain tissue was assessed following the instructions of the TUNEL cell apoptosis detection kit (A113‐01, Vazyme). After deparaffinization with xylene and rehydration through graded ethanol, rat brain paraffin sections underwent heat‐induced antigen retrieval in sodium citrate buffer (pH 6.0) at 95°C–100°C for 20 min. The sections were then incubated with proteinase K (10 μg/mL, P9460, Solarbio) at 37°C for 20 min, followed by two washes with PBS. Non‐specific binding was blocked with 5% donkey serum for 30 min. Subsequently, the sections were incubated overnight at 4°C with NeuN antibody (1:100, bs‐1613R, bioss). After washing, they were incubated with Alexa Fluor 594‐conjugated secondary antibody (1:500, ab150080, Abcam) for 1 h in the dark. The TUNEL working solution was then applied to cover the samples, which were incubated at 37°C in the dark for 1 h. DAPI (C1002, Beyotime) was used to counterstain the nuclei at room temperature for 5 min, followed by two washes with PBS. The apoptosis of neuronal cells in the cerebral cortex was observed under a fluorescence microscope (DM 2500, Leica), with red fluorescence indicating neurons and green fluorescence indicating TUNEL‐positive staining. The apoptotic index was calculated as: (Number of TUNEL^+^ NeuN^+^ cells/Number of NeuN^+^ cells) × 100%.

Following experimental treatments (Control, OGD/R, and OGD/R + JB at 2.5, 5, 10 μM), HAPI cells underwent sequential processing: (1) Dual PBS washes, (2) 4% PFA fixation (30 min), and (3) 0.1% Triton X‐100 permeabilization (5 min). To assess JB's dose‐dependent effects on OGD/R‐triggered microglial apoptosis, TUNEL and DAPI staining were conducted per the manufacturer's protocol.

### 2.11 Enzyme‐Linked Immunosorbent Assay (ELISA)

The right brain tissues of rats were collected for cytokine measurement and Western blot analysis. To quantify inflammatory cytokines (IL‐1β, TNF‐α, IFN‐γ, IL‐1α, IL‐12) in brain tissue, samples were PBS‐washed and homogenized in ice‐cold Tris–HCl buffer with protease inhibitors using a TissueMaster grinder (E6618, Beyotime). After centrifugation at 5000 rpm for 10 min, supernatants were harvested for ELISA analysis. Cytokine levels (IL‐1β, TNF‐α, IFN‐γ, IL‐1α, IL‐12) were determined using commercial ELISA kits (Beyotime: PI301, PT512, PI508, PI561, PI530) following the manufacturer's protocols, with absorbance readings taken at 450 nm. Each sample was tested in 3 independent wells as technical replicates.

HAPI cells were plated in 6‐well plates (6 × 10^5^ cells/well) and treated as described. Following PBS washes, cells were lysed through freeze–thaw cycles. Cell lysates were centrifuged (2000 rpm, 10 min), with supernatants harvested for IL‐1β, TNF‐α, and IFN‐γ level determination.

### 2.12 Flow Cytometry

Microglia were isolated from brain tissues from all experimental groups using the Neural Tissue Dissociation Kits (130‐094‐802, Miltenyi Biotec). Percoll solutions with concentrations of 30% and 70% were prepared, and the microglial cell suspension was added. The samples underwent centrifugation at 800 *g* for 30 min, and the cells located between the 30% and 70% Percoll layers were collected to remove the myelin. HAPI cells were trypsinized (15090046, Invitrogen) for subsequent processing. Microglia were resuspended in PBS (2% FBS), and the cell concentration was adjusted to 1 × 10^7^ cells/mL. Microglia were incubated on ice for 1 h with CD16‐PE antibody (MHCD1604, Invitrogen) and CD206‐APC antibody (17‐2061‐82, Invitrogen). Microglial M1/M2 polarization was assessed by flow cytometry (FACS AriaTM, BD Biosciences) with FlowJo v10 analysis.

Cell apoptosis under various JB treatments and JAK2/STAT3 regulation was analyzed by Annexin V‐APC/PI staining (CA3580, Solarbio) in the OGD/R model. HAPI cells (1 × 10^5^) were pelleted (1000 *g*, 5 min), PBS‐washed, and resuspended in 195 μL Binding Buffer. After adding 5 μL Annexin V‐APC and 10 μL PI, samples were incubated (20 min, dark) before flow cytometric analysis (FlowJo v10).

### 2.13 Lactate Dehydrogenase (LDH) Release Assay

To assess JB's dose‐dependent effects and JAK2/STAT3 regulation on OGD/R‐induced microglial cytotoxicity, HAPI cells (5 × 10^3^ cells/well in 96‐well plates) were treated as described and incubated for 24 h. Following the LDH kit protocol (C0017, Beyotime), cell supernatants were collected after centrifugation. The cell pellets were mixed thoroughly with 150 μL of LDH release reagent and incubated for 1 h. Cell supernatant (120 μL) was reacted with LDH working solution (60 μL) during a 30‐min dark incubation at room temperature. The OD value (490 nm) was determined via microplate spectrophotometry. The cytotoxic effect was quantified using the formula: LDH release rate (%) = [(A_sample_ − A_control_)/(A_standard_ − A_control_)] × 100%.

### 2.14 Immunofluorescence Staining

The brain tissue paraffin sections were heated at 60°C, treated with xylene for dewaxing, and gradually rehydrated using ethanol. Subsequently, antigen retrieval was performed by heating the sections in citrate buffer (pH 6.0) for 2 min, followed by blocking with donkey serum (ANT051, Antgene). HAPI cells on coverslips were fixed using 4% PFA for 20 min, followed by permeabilization with 0.2% Triton X‐100 (diluted in PBS) for 15 min at room temperature. Both the brain tissue sections and HAPI cell coverslips were then blocked with 5% skim milk for 1 h at room temperature before overnight incubation at 4°C with the primary antibodies: Iba‐1 (17198, 1:100, Cell Signaling Technology) and CD206 (PA5‐101657, 1:50, Proteintech), Iba‐1 and CD16 (MA1‐7633, 1:200, Proteintech), CD206 and CD16, and p‐STAT3 (9145, 1:200, Cell Signaling Technology). The following day, the samples were washed three times with PBST and incubated with the corresponding secondary antibodies at 37°C for 2 h. The secondary antibodies used in the study included Alexa Fluor 488 (A‐11094, 1:1000, Invitrogen) and Alexa Fluor 568 (A‐11011, 1:1000, Invitrogen). The nuclei were then treated with DAPI staining for five min, followed by three washing steps before mounting. The immunofluorescence images were captured with a Carl Zeiss Axio Observer 5 fluorescence microscope and subsequently analyzed using ZEN imaging software.

### 2.15 Western Blot

To isolate total protein, rat brain tissue and HAPI cells were lysed with RIPA buffer (P0013C, Beyotime) supplemented with protease and phosphatase inhibitors. Following sonication (15 min, 4°C) and centrifugation (12,000 rpm, 30 min), the supernatant was collected, and protein levels were measured with a BCA assay kit (P0012, Beyotime). Proteins were then resolved on 10% SDS‐PAGE gels and electroblotted onto polyvinylidene fluoride (PVDF) membranes (03010040001, Merck Millipore). The membranes were treated with 5% skim milk for 1 h at room temperature to block nonspecific binding. After washing, they were exposed to the designated primary antibodies and kept at 4°C overnight: iNOS (ab178945, 1:100, Abcam), CD16 (ab223200, 1:500, Abcam), Arg‐1 (sc‐81154, 1:200, Santa Cruz Biotechnology), CD206 (PA5‐101657, 1:1000, Proteintech), p‐STAT3 (Ser727) (PSTAT3‐340AP, 1:1000, Invitrogen), p‐STAT3 (Tyr705) (orb615050, 1:1000, Biorbyt), STAT3 (PA5‐120138, 1:1000, Invitrogen), JAK2 (3230S, 1:1000, Cell Signaling Technology), p‐JAK2 (Tyr1007/1008) (WL02997, 1:1000, Wanleibio), β‐actin (4970T, 1:2000, Cell Signaling Technology), Caspase‐3 (9662T, 1:1000, Cell Signaling Technology), Cleaved‐Caspase‐3 (9661T, 1:1000, Cell Signaling Technology), Caspase‐9 (PA5‐22252, 1:1000, Invitrogen), and Cleaved‐Caspase‐9 (9509T, 1:1000, Cell Signaling Technology). Then the membranes were treated with a secondary antibody (ab205718, 1:2000, Abcam) for 2 h at room temperature. After incubation, protein bands were detected using an ECL chemiluminescence kit (P0018AS, Beyotime) and imaged with a ChemiDoc system (Bio‐Rad). Band intensity was analyzed using ImageJ software for quantification.

### 2.16 Statistical Analysis

Statistical analysis was performed in GraphPad Prism 9, with results reported as mean ± SD. In this study, the normality of all data was assessed using the Shapiro–Wilk test. If the data conformed to a normal distribution, intergroup differences were calculated using ANOVA followed by Tukey's post hoc test. A *p*‐value below 0.05 indicated significance.

## 3 Results

### 3.1 JB Alleviates Neurological Dysfunction and Pathological Damage Induced by CIRI in MCAO/R Rats

**FIGURE 1:**
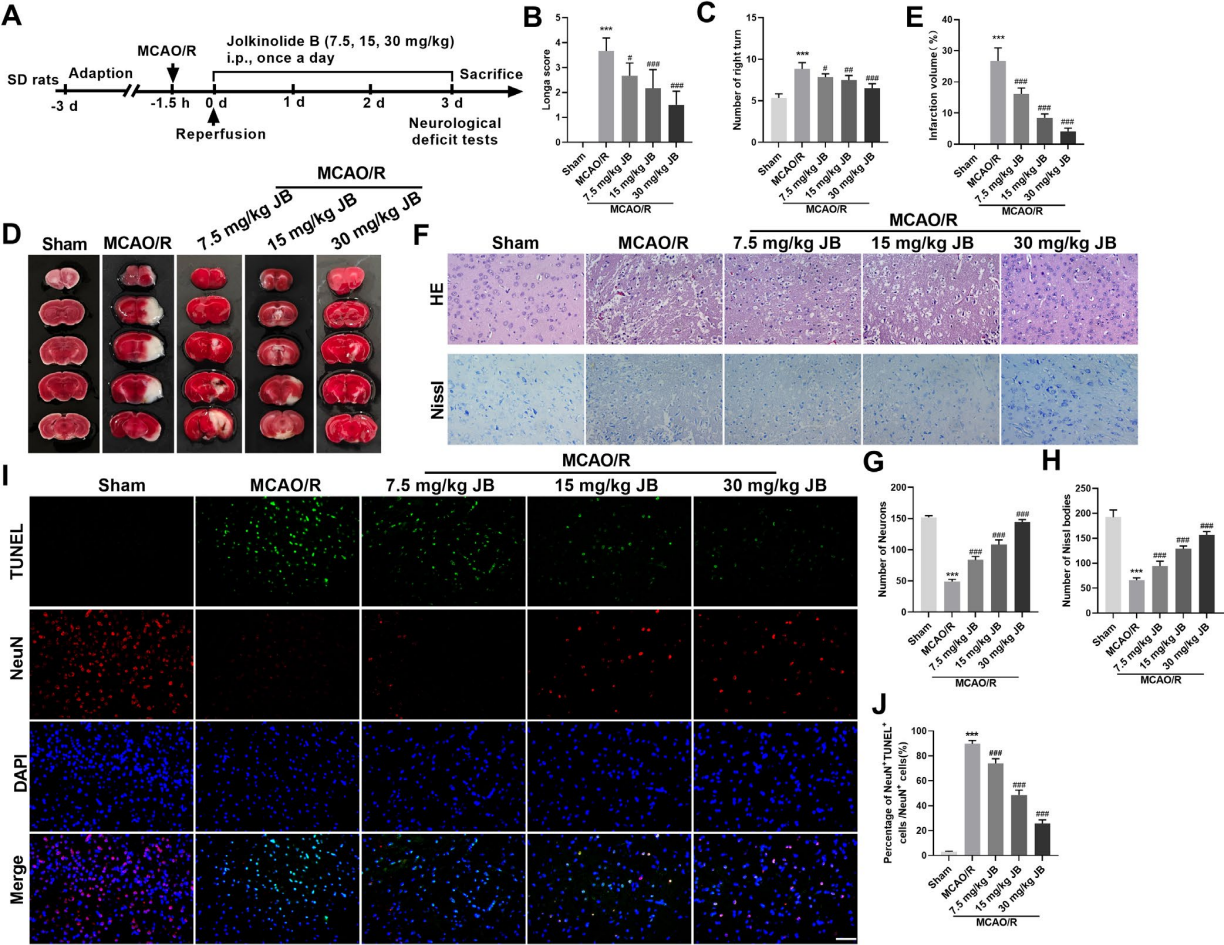
JB alleviates neurological dysfunction and pathological damage induced by CIRI in MCAO/R rats. (A) Schematic diagram of the experimental workflow. Rats in each group received intraperitoneal injections of JB once daily for 3 consecutive days starting from the onset of reperfusion. Neurological behavior tests were performed 1 h after the final administration. Subsequently, the animals were sacrificed, and brain tissues were rapidly harvested for further analysis. (B, C) Neurological function deficits were assessed using the Longa scoring system and the corner test. *n* = 18. (D, E) TTC staining was used to evaluate the extent of cerebral infarction and measure the infarct volume in each group. (F–H) HE staining was performed to observe pathological changes in cortical tissue structures across groups. Nissl staining was used to quantify the number of Nissl bodies. Scale bar 50 μm. (I, J) TUNEL staining was conducted to detect neuronal apoptosis in the cerebral cortex. Scale bar 50 μm. *n* = 6. ***p < 0.001 vs. sham; ^#^ *p* < 0.05, ^##^ *p* < 0.01, ^###^ *p* < 0.001 vs. MCAO/R.

To examine the potential neuroprotective effects of JB, a MCAO/R model was utilized. JB was administered intraperitoneally at varying doses (7.5, 15, and 30 mg/kg) beginning at reperfusion onset, with daily injections for three consecutive days. On the third day, Longa scoring and the Corner test were performed. Neurological impairment, as assessed by deficit scores and corner turning behavior, was significantly greater in the MCAO/R group relative to sham‐operated animals. In contrast, JB treatment significantly reduced both Longa scores and corner turning frequencies compared to the MCAO/R group (Figure 1B,C). TTC staining results indicated a significant reduction in infarct volume with JB treatment versus the MCAO/R model (Figure 1D,E). HE staining demonstrated a marked reduction in cortical neuron count, along with irregular morphology and nuclear shrinkage, in the MCAO/R group. After JB administration, the number of cortical neurons increased, the cell morphology appeared relatively intact, nucleoli were prominent, and nuclear shrinkage was alleviated (Figure 1F,G). In the MCAO/R group, Nissl staining demonstrated that cortical neurons showed somatic swelling, nuclear shift, and irregular dispersion, accompanied by partial Nissl body breakdown leading to translucent patches, reduced staining density, and a notable decline in Nissl body numbers. JB treatment alleviated structural damage to cortical neurons and increased the number of Nissl bodies (Figure 1F,H). TUNEL staining demonstrated that JB significantly reduced the apoptosis rate in the cortical tissue of MCAO/R rats (Figure 1I,J). These findings indicate that JB treatment alleviates neurological deficits and pathological changes in brain tissue, while also mitigating neuronal injury in rats with MCAO/R, and the effects are dose‐dependent.

### 3.2 JB Attenuates Neuroinflammation and Enhances Microglial M2 Polarization in MCAO/R Rat Brains

**FIGURE 2:**
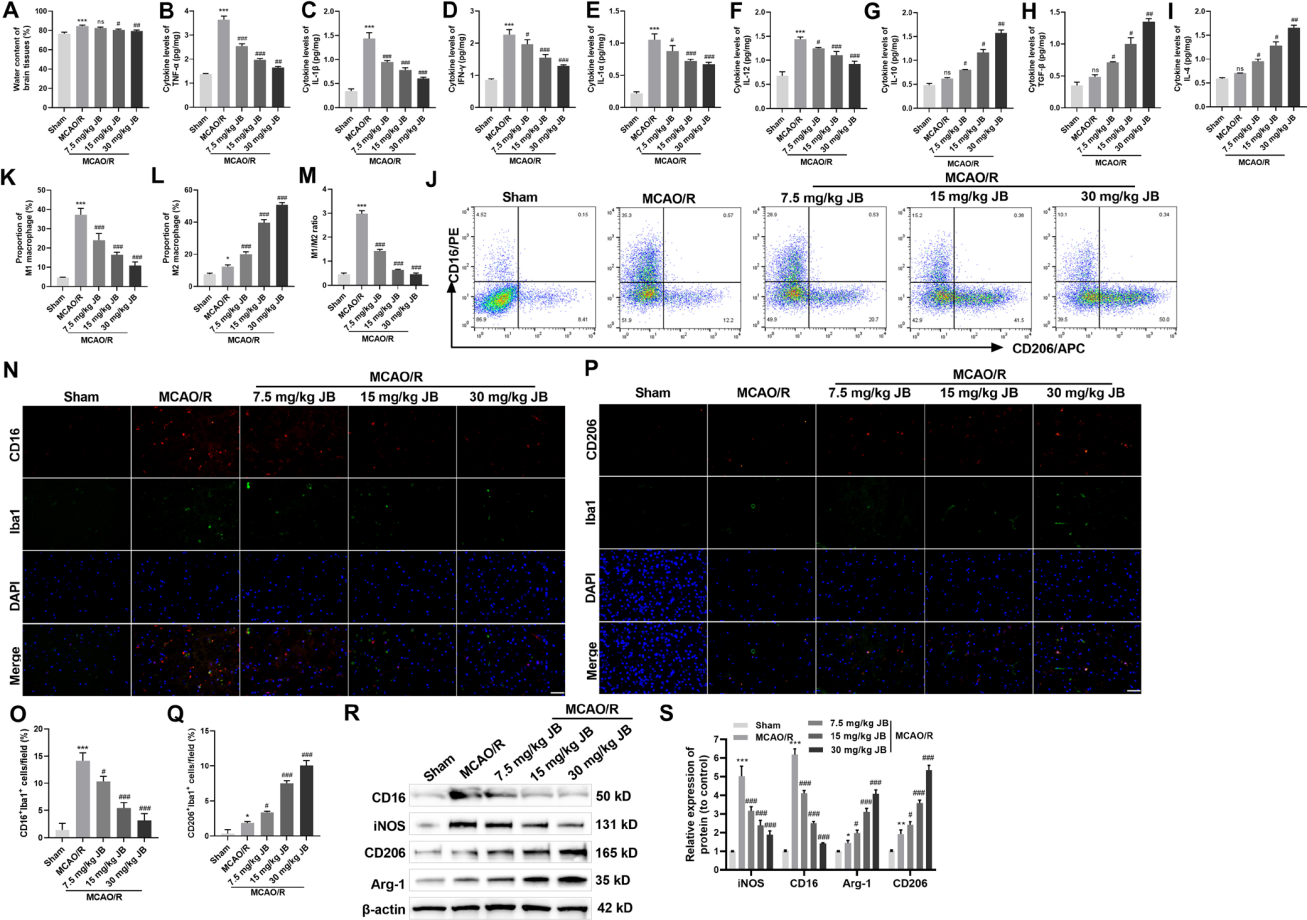
JB reduces inflammatory injury in the brain tissue of MCAO/R rats and promotes M2 polarization of microglia. (A) The effect of JB on brain water content (%) in MCAO/R rats. (B–I) ELISA was used to assess the effects of JB on the levels of pro‐inflammatory cytokines (TNF‐α, IL‐1β, IFN‐γ, IL‐1α, IL‐12) and anti‐inflammatory cytokines (IL‐10, TGF‐β, and IL‐4) in the brain tissue of MCAO/R rats. (J–M) Flow cytometry was performed to evaluate the effect of JB on the proportions of M1‐type (CD16^+^) and M2‐type (CD206^+^) microglia in the brain tissue of MCAO/R rats. (N–Q) Immunofluorescence was used to detect the effect of JB on the proportion of M1‐ and M2‐type microglia in the brain tissue of MCAO/R rats. CD16^+^/Iba1^+^ cells were defined as M1‐type microglia, and CD206^+^/Iba1^+^ cells were defined as M2‐type microglia. Scale bar 50 μm. (R, S) Western blot analysis was performed to determine the effects of 7.5, 15, and 30 mg/kg JB on the protein expression levels of M1 markers (iNOS, CD16) and M2 markers (Arg‐1, CD206) in microglia from the brain tissue of MCAO/R rats. *n* = 6. *p < 0.05, **p < 0.01, ***p < 0.001 vs. sham; ^#^ *p* < 0.05, ^###^ *p* < 0.001 vs. MCAO/R.

CIRI can lead to the inactivation of ion channels on neuronal cell membranes, promoting the influx of calcium and sodium ions, resulting in neuronal swelling and subsequent brain edema, which is manifested as increased brain water content [25]. Quantitative analysis revealed a significant elevation in brain water content following MCAO/R versus sham operation, with JB therapy demonstrating a dose‐responsive reduction of cerebral edema (Figure 2A). Under conditions of neuroinflammatory injury, microglia are rapidly activated and categorized into functional phenotypes: microglia polarized to the M1 state (CD16^+^) exhibiting characteristic secretion of pro‐inflammatory factors like TNF‐α, interleukin‐6 and interleukin‐1β, thereby exacerbating the inflammatory response; and anti‐inflammatory M2 microglia (CD206^+^), which release anti‐inflammatory factors such as Arg‐1, IL‐10, TGF‐β, and IL‐4, thereby alleviating local inflammation [26, 27]. ELISA results showed that, compared with the sham group, the levels of pro‐inflammatory cytokines (IL‐1β, TNF‐ɑ, IFN‐γ, IL‐1α, and IL‐12) in the brain tissue of MCAO/R rats were significantly increased, while the levels of anti‐inflammatory cytokines (IL‐10, TGF‐β, and IL‐4) were significantly decreased. Intraperitoneal injection of JB reduced the levels of pro‐inflammatory cytokines and increased the levels of anti‐inflammatory cytokines (Figure 2B–I). Flow cytometry results indicated that after brain tissue injury, both M1 (CD16^+^) and M2 (CD206^+^) microglia increased. Following JB intervention, CD16^+^ microglia populations (M1) were markedly reduced, with parallel increases in CD206^+^ cells (M2) and their relative proportion (Figure 2J–M). Collectively, the data reveal that JB exerts dual effects by reducing cerebral inflammatory injury and promoting M2 phenotype polarization of microglia in MCAO/R rats.

To assess JB's effects on microglial phenotypes, we performed dual immunofluorescence staining using: (i) CD16 with Iba‐1 for M1 identification, and (ii) CD206 with Iba‐1 for M2 detection in cerebral cortical sections (Figure 2N–Q). Relative to sham‐operated controls, both M1 microglia and M2 microglia were significantly increased in the MCAO/R group. After JB treatment, the proportion of M1 microglia in MCAO/R rats significantly decreased, while the proportion of M2 microglia markedly increased. Western blot analysis quantified phenotypic markers: pro‐inflammatory M1 microglial markers (iNOS, CD16) and anti‐inflammatory M2 microglial markers (Arg‐1, CD206) in cerebral tissue. Quantitative analysis revealed elevated expression of both M1 markers (iNOS, CD16) and M2 markers (Arg‐1, CD206) in MCAO/R versus sham controls. JB treatment dose‐dependently reversed these changes, suppressing M1 markers while enhancing M2 markers (Figure 2R,S). These findings further demonstrated that JB‐mediated neuroprotection in CIRI involves microglial phenotypic conversion, from M1‐dominant neuroinflammation to M2‐predominant.

### 3.3 JB Blocks JAK2/STAT3 Signaling in MCAO/R Rats

**FIGURE 3:**
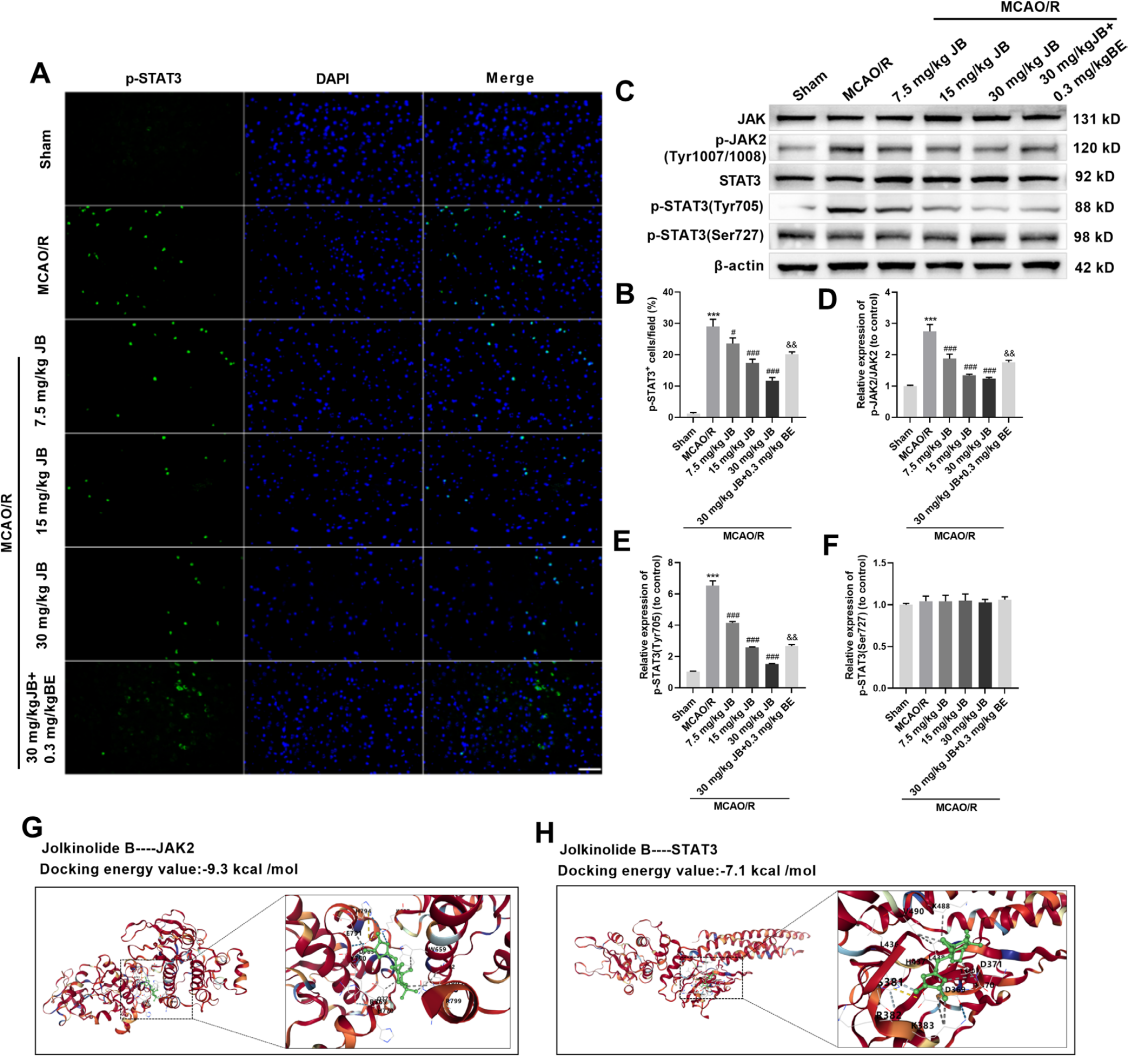
JB blocks JAK2/STAT3 signaling in MCAO/R rats. (A, B) The effect of JB (7.5, 15, 30 mg/kg) on p‐STAT3 nuclear translocation in MCAO/R rats was analyzed by immunofluorescence. Scale bar 50 μm. (C–F) The effects of JB (7.5, 15, 30 mg/kg) on JAK2/STAT3 pathway proteins (JAK2, p‐JAK2, STAT3, p‐STAT3 Tyr705/Ser727) in MCAO/R rat brains were analyzed by Western blot. (G, H) Molecular docking of JB with JAK2 and JB with STAT3. *n* = 6. ***p < 0.001 vs. sham; ^#^ *p* < 0.05, ^###^ *p* < 0.001 vs. MCAO/R, ^&&^ *p* < 0.01 vs. MCAO/R + 30 mg/kg JB.

To further investigate the regulatory effect of JB on the JAK2/STAT3 signaling pathway, immunofluorescence staining and Western blot analysis were used to detect the expression changes of key signaling molecules. Phosphorylated STAT3 (p‐STAT3) undergoes nuclear translocation upon activation. Immunofluorescence imaging demonstrated pronounced nuclear accumulation of p‐STAT3 in MCAO/R brains, and the proportion of p‐STAT3‐positive cells increased, contrasting with minimal staining in sham controls, while JB treatment dose‐dependently reduced its expression, indicating that JB can inhibit STAT3 phosphorylation (Figure 3A,B). Western blot analysis demonstrated a marked upregulation of p‐STAT3 (Tyr705) and p‐JAK2 in MCAO/R rats compared to sham controls, further confirming that brain injury activates the JAK2/STAT3 signaling pathway. JB administration suppressed p‐STAT3 (Tyr705) levels and decreased the p‐JAK2/JAK2 ratio in a dose‐dependent manner, indicating targeted modulation of JAK2‐STAT3 signaling in MCAO/R rats. It is noteworthy that co‐treatment with JB and the JAK2 activator BE increased the expression level of p‐STAT3 as well as the phosphorylation levels of JAK2 and STAT3 (Tyr705). Additionally, JB exhibited distinct phospho‐site selectivity, potently inhibiting JAK2 (Tyr1007/1008) and STAT3 (Tyr705) phosphorylation without affecting the Ser727 site of STAT3 (Figure 3C–F). The binding energy between JB and JAK2 was −9.3 kcal/mol, and that between JB and STAT3 was −7.1 kcal/mol, indicating that JB can bind well to both JAK2 and STAT3 molecules (Figure 3G,H), further supporting its role via the JAK2/STAT3 pathway. These results suggest that JB exerts neuroprotective effects by inhibiting the activation of the JAK2/STAT3 signaling pathway.

### 3.4 JB Improved HAPI Cell Survival and Decreased Apoptosis Under OGD/R Conditions

**FIGURE 4:**
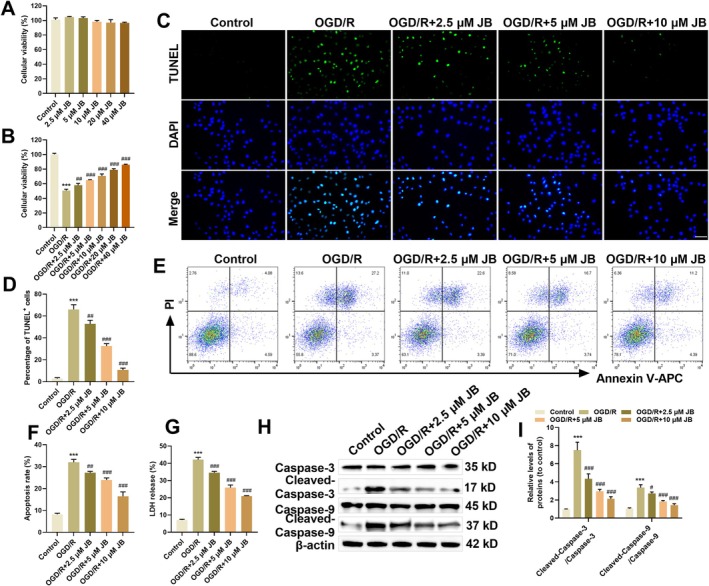
JB improved HAPI cell survival and decreased apoptosis under OGD/R conditions. (A) CCK‐8 assay was used to assess JB (0, 2.5, 5, 10, 20, 40 μM) effects on HAPI cell viability. (B) CCK‐8 assay determined the effects of JB (0, 2.5, 5, 10, 20, 40 μM) on the viability of OGD/R‐treated HAPI cells. (C, D) TUNEL assay to determine the effects of JB at concentrations of 2.5, 5, and 10 μM on apoptosis in OGD/R‐treated HAPI cells. Scale bar 50 μm. (E, F) Flow cytometry measured apoptosis rates in OGD/R‐injured HAPI cells after JB treatment (2.5, 5, 10 μM). (G) LDH release assay to measure the effects of JB at concentrations of 2.5, 5, and 10 μM on LDH release in OGD/R‐treated HAPI cells. (H, I) Western blot analysis of the effects of JB (2.5, 5, 10 μM) on the expression of apoptosis‐related proteins (Caspase‐3, Cleaved‐Caspase‐3, Caspase‐9, Cleaved‐Caspase‐9) in OGD/R‐treated HAPI cells. *n* = 6. ***p < 0.001 vs. Control; ^#^ *p* < 0.05, ^##^ *p* < 0.05, ^###^ *p* < 0.001 vs. OGD/R.

JB treatment (2.5, 5, 10, 20, 40 μM) showed no significant impact on HAPI cell viability in CCK‐8 assays. However, JB at 2.5 and 5 μM slightly increased cell viability, while higher concentrations of JB (10, 20, 40 μM) reduced viability (Figure 4A). HAPI cell viability significantly dropped after post‐OGD/R. The addition of JB at different concentrations significantly improved cell viability in a dose‐dependent manner (Figure 4B). Considering the safety of JB's effects, concentrations of 2.5, 5, and 10 μM were selected for subsequent experiments. The protective role of JB against OGD/R damage was evaluated by analyzing apoptosis levels in HAPI microglial cells. TUNEL staining and flow cytometry analysis showed that OGD/R exposure markedly elevated HAPI cell apoptosis versus controls, while JB treatment effectively suppressed apoptosis (Figure 4C–F). Assessment of LDH release, a well‐established marker of membrane integrity and cytotoxicity, revealed significantly higher levels in OGD/R‐exposed HAPI cells versus controls. JB treatment effectively attenuated this OGD/R‐induced LDH release (Figure 4G). These findings indicate that JB not only exhibits no significant cytotoxicity to primary microglial cells but also promotes HAPI cell viability and inhibits apoptosis under OGD/R conditions.

During the process of apoptosis, Caspase‐9 acquires catalytic activity by activating the apoptosome, which subsequently activates the downstream effector protein Caspase‐3, ultimately leading to the complete execution of cell apoptosis [28, 29]. Western blot analysis revealed elevated Cleaved‐Caspase‐3/Caspase‐3 and Cleaved‐Caspase‐9/Caspase‐9 ratios in HAPI cells subjected to OGD/R, indicating that the intrinsic apoptotic pathway was activated. JB treatment reduced these ratios, and the inhibitory effect became more pronounced with increasing JB concentrations (Figure 4H,I). These findings further confirmed that JB can suppress OGD/R‐induced apoptosis in HAPI cells, potentially by mitigating apoptosis levels through the inhibition of cleavage of apoptosis‐related proteins.

### 3.5 JB Protects HAPI Cells From OGD/R‐Triggered Inflammatory Damage via Suppression of JAK2/STAT3 Pathway Activation

**FIGURE 5:**
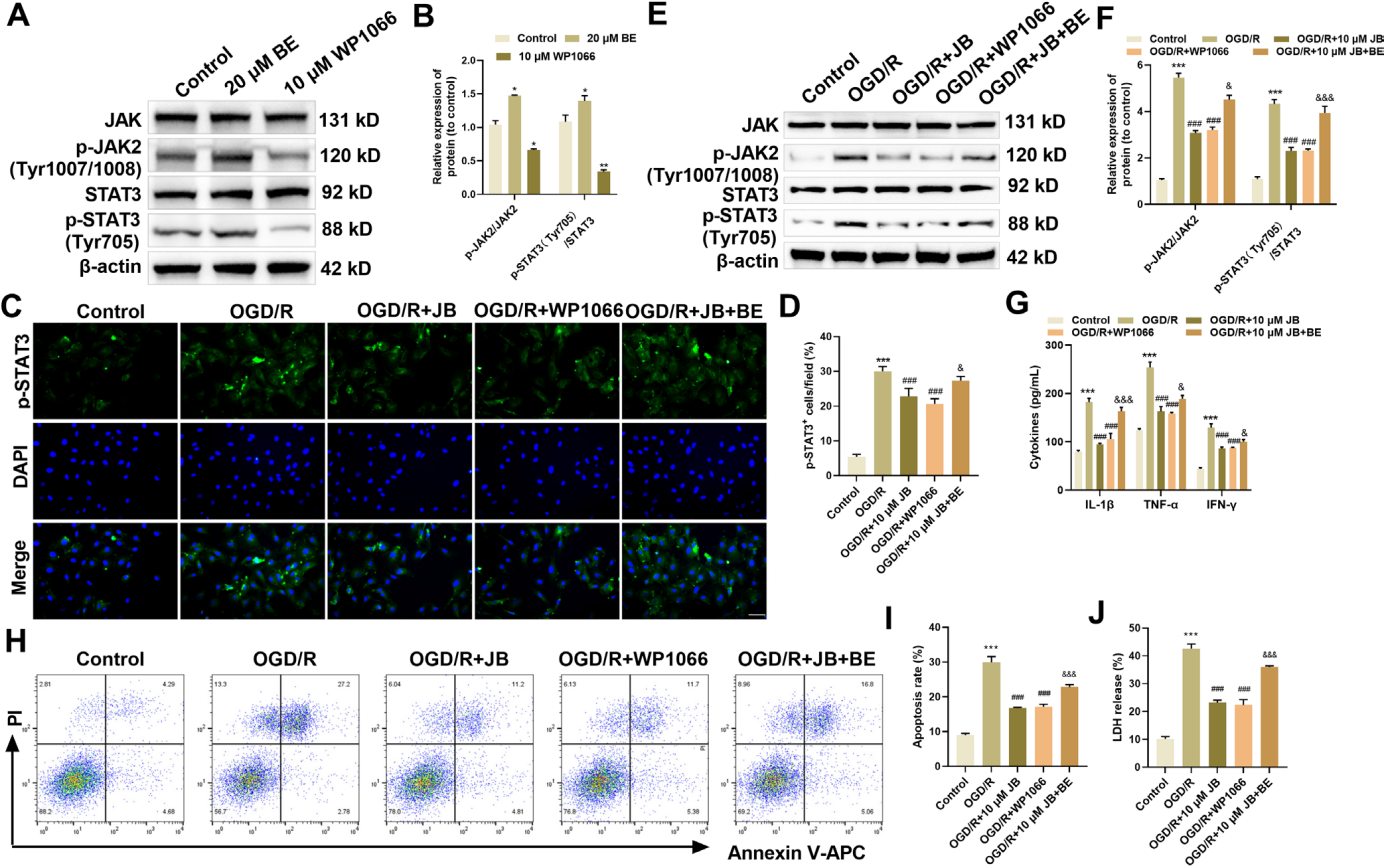
JB protects HAPI cells from OGD/R‐triggered inflammatory damage via suppression of JAK2/STAT3 pathway activation. (A, B) Western blot was used to verify the effects of JAK2 activator and inhibitor. (C, D) Immunofluorescence analysis was conducted to examine the effects of JB, WP1066, and BE on the nuclear translocation of p‐STAT3 in OGD/R‐treated HAPI cells. Scale bar 50 μm. (E, F) Western blot assessed JB, WP1066, and BE effects on JAK2/STAT3 pathway proteins (JAK2, p‐JAK2, STAT3, p‐STAT3) in OGD/R‐treated HAPI cells. (G) ELISA was used to assess the effects of JB, WP1066, and BE on the levels of pro‐inflammatory cytokines (IL‐1β, TNF‐α, and IFN‐γ) in OGD/R‐treated HAPI cells. (H, I) Flow cytometry was conducted to analyze the effects of JB, WP1066, and BE on the apoptosis rate of OGD/R‐treated HAPI cells. (J) LDH release assay assessed JB, WP1066, and BE effects on OGD/R‐treated microglial cells. *n* = 6. *p < 0.05, **p < 0.01, ***p < 0.001 vs. Control; ^###^ *p* < 0.001 vs. OGD/R; ^&^ *p* < 0.05, ^&&&^ *p* < 0.001 vs. OGD/R + JB.

WP1066, as a specific JAK2 inhibitor, can simultaneously downregulate JAK2 protein expression and inhibit JAK2 phosphorylation, while BE significantly activates the JAK2/STAT3 signaling pathway [30, 31]. This study utilized WP1066 (a JAK2 inhibitor) and BE (a JAK2 activator) to elucidate JB's protective mechanism against OGD/R‐induced inflammatory injury in microglia via the JAK2/STAT3 signaling pathway. Western blot results showed that treatment with the JAK2 activator (BE) significantly increased the phosphorylation levels of JAK2 and STAT3 in the cells (Figure 5A,B). In contrast, treatment with the JAK2 inhibitor WP1066 significantly decreased the phosphorylation levels, confirming that BE and WP1066 can effectively activate and inhibit the JAK2/STAT3 signaling pathway, respectively. Immunofluorescence results showed that OGD/R induced STAT3 phosphorylation, demonstrating enhanced nuclear translocation of p‐STAT3 in microglial cells. Treatment with JB or WP1066 significantly reduced p‐STAT3 expression, while co‐treatment with BE and JB significantly increased p‐STAT3 expression compared to the OGD/R + JB group (Figure 5C,D). Protein expression analysis showed that OGD/R stimulation markedly elevated the phosphorylation levels of both JAK2 and STAT3 (Tyr705) in HAPI cells. These ratios were reduced following treatment with JB or WP1066. However, combined BE/JB treatment elevated p‐JAK2/JAK2 and p‐STAT3(Tyr705)/STAT3 ratios relative to JB monotherapy in OGD/R conditions (Figure 5E,F). These findings indicate that JB inhibits the phosphorylation of JAK2 and STAT3, with an inhibitory effect comparable to WP1066, while BE attenuates the inhibitory effect of JB on the JAK2/STAT3 signaling pathway.

ELISA analysis revealed OGD/R‐induced upregulation of IL‐1β, TNF‐α, and IFN‐γ in HAPI cells. Both JB and WP1066 (JAK2 inhibitor) effectively attenuated this inflammatory response, while BE (JAK2 activator) counteracted JB's suppressive effects (Figure 5G). Further analysis using flow cytometry and LDH release assays confirmed that JB and WP1066 treatment significantly reduced OGD/R‐induced apoptosis rates and LDH release levels, while BE treatment partially reversed the cytoprotective effects of JB (Figure 5H–J). Collectively, JB appears to alleviate microglial dysfunction through JAK2/STAT3 blockade, diminishing both inflammatory and apoptotic responses.

### 3.6 JB Suppresses JAK2/STAT3 Activation and Induces M2 Phenotype Transition in OGD/R‐Injured Microglia

**FIGURE 6:**
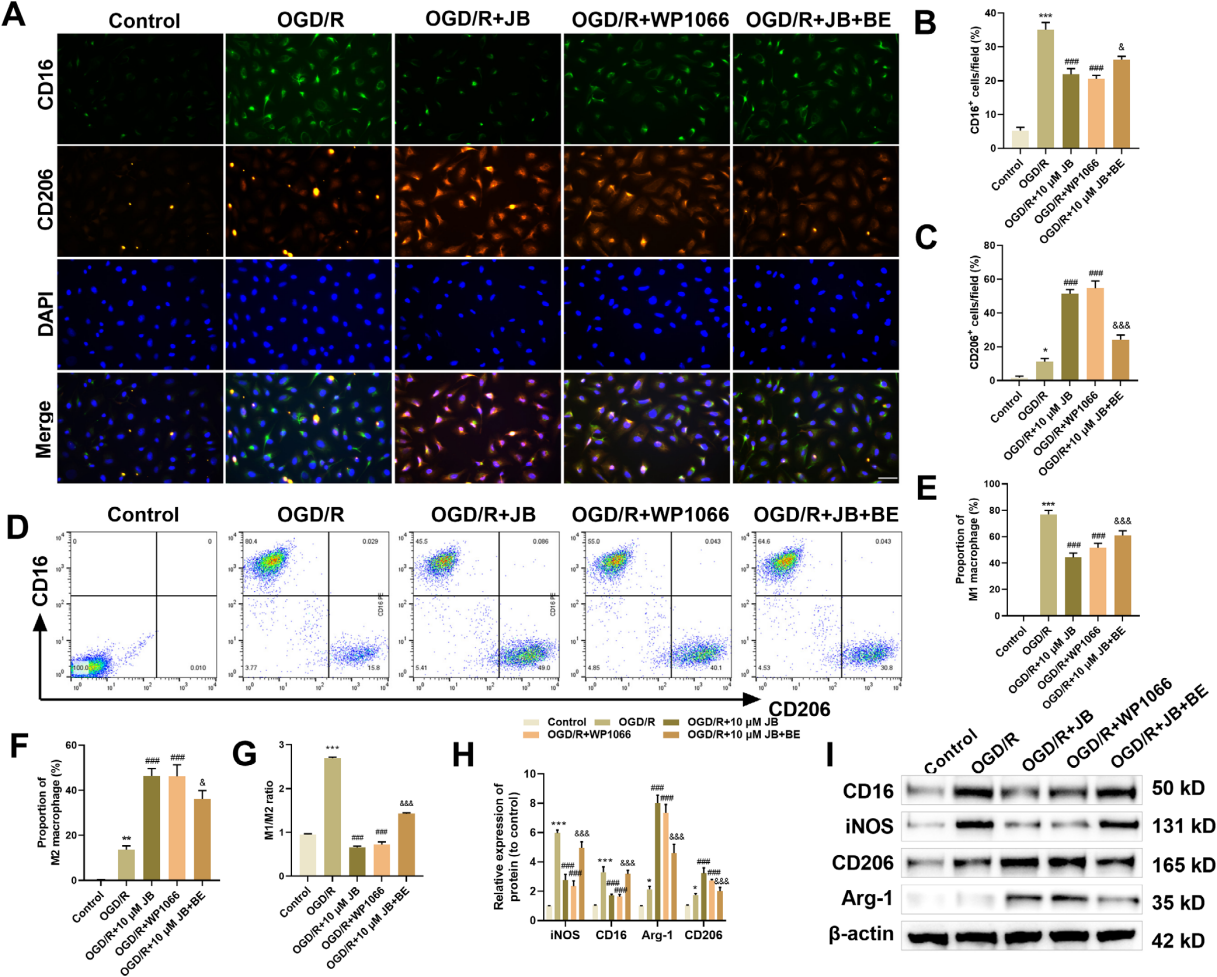
JB suppresses JAK2/STAT3 activation and induces M2 phenotype transition in OGD/R‐injured microglia. (A–C) Immunofluorescence analysis to assess the effects of JB, WP1066, and BE on the polarization state of OGD/R‐treated HAPI cells by quantifying the expression levels of M1 markers (CD16^+^) and M2 markers (CD206^+^). Scale bar 50 μm. (D–G) Flow cytometry was performed to determine the effects of JB, WP1066, and BE on the proportions of M1 and M2 microglia in OGD/R‐treated HAPI cells. (H, I) Western blot measured JB, WP1066, and BE modulation of M1 (iNOS, CD16) and M2 (Arg‐1, CD206) marker expression in the OGD/R HAPI model. *n* = 6. *p < 0.05, **p < 0.01, ***p < 0.001 vs. Control; ^###^ *p* < 0.001 vs. OGD/R; ^&^ *p* < 0.05, ^&&&^ *p* < 0.001 vs. OGD/R + JB.

To elucidate JB's modulation of JAK2/STAT3‐dependent microglial polarization, we quantified M1 (iNOS, CD16) and M2 (Arg‐1, CD206) markers through multi‐method analysis. Both immunofluorescence and flow cytometry revealed OGD/R‐induced elevation of M1 (CD16^+^) and M2 (CD206^+^) HAPI cell populations. JB or WP1066 treatment significantly decreased the proportion of M1 HAPI cells while enhancing the proportion of M2 HAPI cells. However, co‐treatment with the JAK2 activator BE significantly increased M1 HAPI cells and decreased M2 HAPI cells compared to JB treatment alone (Figure 6A–G). Western blot detection indicated that OGD/R stimulation increased the protein levels of microglial activation markers, with both M1‐type (iNOS, CD16) and M2‐type (Arg‐1, CD206) markers being upregulated. Both treatments suppressed M1 phenotype markers (iNOS, CD16) and enhanced M2‐associated factors (Arg‐1, CD206). Co‐treatment with BE and JB significantly reversed the regulatory effects of JB on M1/M2 markers in HAPI cells (Figure 6H,I). These results indicate that JB can shift microglial polarization from M1 to M2 via JAK2/STAT3 inhibition, while the JAK2 activator partially reverses this regulatory effect.

## 4 Discussion

CIRI represents a crucial pathological mechanism in IS, involving multifaceted processes such as neuroinflammation, redox imbalance, programmed cell death, bioenergetic failure, and cerebrovascular compromise [32, 33, 34]. Despite the unique advantages of active components from natural products in alleviating CIRI, including reducing neuroinflammation and decreasing reactive oxygen species (ROS) production [35], treatment drugs with defined molecular actions are still lacking in clinical practice. JB, as a natural compound with significant biological activity, holds potential application value in anti‐inflammatory, anti‐tumor, and neuroprotective fields [36, 37, 38]. The present study demonstrated that JB treatment effectively attenuated CIRI, as manifested by remarkable improvements in neurological function, substantial reduction in cerebral infarction volume, decreased neuronal loss in cortical regions, and significant suppression of cortical neuronal apoptosis. In addition, in vitro experiments demonstrated that JB not only significantly enhanced the viability of OGD/R‐treated microglial cells but also exerted anti‐apoptotic effects by inhibiting the mitochondrial apoptotic pathway, reducing the expression of key apoptotic markers, including Cleaved‐Caspase‐3 and Cleaved‐Caspase‐9. This indicates that JB can not only effectively alleviate primary neuronal injury caused by ischemia–reperfusion but also significantly improve the secondary injury process, highlighting its potential as a promising therapeutic candidate.

Neuroimmune inflammation is a critical component of the pathophysiology of IS and a major target for the development of new therapies [5]. Under pathological conditions, microglia quickly become activated and differentiate into two functional phenotypes—the pro‐inflammatory M1 type and the anti‐inflammatory M2 type [39, 40]. Published findings reveal M1 microglia, in the state of classical activation, exhibit strong phagocytic and cytotoxic effects. Upon polarization, they promote the secretion and synthesis of inflammatory mediators such as IL‐1β, IL‐6, and IFN‐γ, as well as iNOS and ROS, leading to neuronal dysfunction, damage, and degeneration. M2 polarization plays a role in promoting wound healing, immune regulation, and tissue repair in various central nervous system diseases. Anti‐inflammatory factors such as CD206, IL‐10, TGF‐β, IL‐4, and IGF‐1 are upregulated, thereby modulating inflammatory processes and promoting recovery [41]. After central nervous system injury, most activated microglia at the injury site exhibit M2 polarization, promoting repair. However, approximately one week after the injury, microglia transition to the M1 phenotype, inducing the production of pro‐inflammatory signaling molecules, thereby aggravating neural damage [42]. This dual characteristic of neuroprotection and neurotoxicity makes microglia a critical target for mitigating brain neuronal damage in stroke treatment. This study demonstrated that JB exerts significant regulatory effects on microglial polarization by inhibiting JAK2/STAT3 signal transduction. In the MCAO/R model, JB not only alleviated brain edema and reduced pro‐inflammatory cytokine levels but, more importantly, significantly improved the M1/M2 polarization balance by diminishing M1‐type (CD16^+^/Iba‐1^+^) cells and augmenting M2‐type (CD206^+^/Iba‐1^+^) cells. This effect was further validated in the OGD/R‐induced microglial inflammation model. Additionally, the JAK2 inhibitor WP1066 exhibited synergistic effects with JB, both significantly promoting microglial M2 polarization. In contrast, BE treatment (a JAK2 agonist) eliminated the therapeutic benefits of JB. These corroborative results indicate that JB promotes M2 polarization of microglia by inhibiting the JAK2/STAT3 signaling axis. It is noteworthy that after CIRI, microglia undergo bidirectional polarization, meaning that both the pro‐inflammatory M1 phenotype and the anti‐inflammatory, reparative M2 phenotype are simultaneously activated [43]. We observed that the levels of M2 markers (Arg‐1, CD206) in the brain tissue of rats in the MCAO/R group were higher than those in the sham group, which is highly consistent with the findings of Cheng et al. [44]. This may be due to the initiation of endogenous protective and repair responses by the body after injury, with M2‐type microglia being mobilized to exert anti‐inflammatory and tissue repair functions. Furthermore, JB promotes M2 polarization of microglia and reduces apoptosis of M2 cells, thereby increasing their survival rate. These two effects may have a synergistic effect: on the one hand, JB promotes M2 polarization and increases the proportion of the M2 phenotype; on the other hand, JB inhibits apoptosis of M2 cells, further enhancing their survival.

The JAK/STAT pathway comprises three core components: membrane‐bound tyrosine kinase receptors, intracellular JAK responsible for signal transduction, and STAT that mediates downstream responses. This pathway is involved in the regulatory processes of various cytokines and growth factors [45, 46]. The pathway participates in diverse biological functions, encompassing the control of cell division, movement, apoptotic processes, and the maintenance of immune cells [47, 48]. Extensive research has established that the JAK2/STAT3 signaling serves as a central regulatory hub for microglial polarization. Its activation state can significantly influence the phenotypic transformation of microglia, thereby modulating the progression of neuroinflammatory responses. This pathway plays a critical role in the pathological progression and tissue repair of neurological diseases. According to Chen et al., increased levels of miR‐204‐5p in the ventromedial prefrontal cortex (vmPFC) of chronic unpredictable mild stress (CUMS) rats significantly inhibited the JAK2/STAT3 signaling pathway, induced microglial anti‐inflammatory and neuroprotective effects, and improved neuronal injury [49]. Zhong et al. employed genetic and ischemic models to demonstrate STAT3's central role in JAK2/STAT3 signaling. Their work showed that STAT3 knockout disrupts pathway function, promotes neuroprotective M2 microglial polarization, and mitigates cerebral ischemic damage [50]. Our investigation demonstrated that JB inhibited the phosphorylation of JAK2 and STAT3, thereby blocking the JAK2/STAT3 signaling pathway, promoting M2 polarization of microglia, and alleviating ischemia–reperfusion injury. Furthermore, in the OGD/R microglia in vitro experiments, the JAK2 inhibitor WP1066 downregulated the expression level of p‐STAT3, whereas the JAK2 activator BE attenuated the inhibitory effect of JB on p‐STAT3 expression. These findings indicate that STAT3 phosphorylation occurs downstream of p‐JAK2. Studies by Wei et al. revealed EPO‐induced phosphorylation of both JAK2 and STAT3, with AZD1480 selectively inhibiting the STAT3 activation cascade [51]. Schust et al. demonstrated that Stattic inhibits the binding of STAT3 to the SH2 domain of kinases, thereby preventing the phosphorylation and activation of STAT3, while the phosphorylation of JAK2 was not significantly reduced [52]. These findings further support that STAT3 phosphorylation occurs downstream of p‐JAK2.

STAT3 activation depends on coordinated phosphorylation at both Tyr705 and Ser727 residues [53], which are regulated by different upstream kinases. Specifically, Tyr705 phosphorylation is primarily mediated by JAK2 [54, 55]. Conversely, Ser727 phosphorylation is controlled by independent Protein Kinase C (PKC) and Mitogen‐Activated Protein Kinase (MAPK) signaling pathways [56, 57]. Our results demonstrate that JB effectively suppresses MCAO/R‐induced STAT3 Tyr705 phosphorylation and nuclear translocation, without significantly altering Ser727 phosphorylation status. This suggests that JB may act as a selective JAK2 inhibitor, preferentially blocking JAK2‐mediated Tyr705 phosphorylation and subsequent nuclear translocation, with minimal impact on Ser727 modifications mediated by other kinases. Such selective inhibition indicates that JB may regulate microglial polarization by precisely targeting the JAK2‐STAT3 (Tyr705) axis, thereby avoiding interference with other regulatory pathways of STAT3 to some extent.

Although this study confirmed that JB exerts significant neuroprotective effects against CIRI by regulating the JAK2/STAT3 signaling pathway and microglial M1/M2 polarization, there are still some limitations that need to be addressed in future research. This study demonstrated that JB binds well to JAK2 and STAT3 through molecular docking, and verified the inhibitory effect of JB on the JAK2/STAT3 pathway using JAK2 inhibitors and activators. However, the direct molecular targets of JB were not thoroughly investigated. Future studies should further clarify whether JB directly and physically interacts with key proteins in the JAK2/STAT3 signaling pathway (such as JAK2 or STAT3), in order to more precisely elucidate its mechanism of action. Although the focus of this study was on the JAK2/STAT3 pathway, we cannot completely rule out the possibility that JB exerts its anti‐inflammatory effects via other signaling pathways. Previous literature has reported that JB may also have inhibitory effects on other key inflammatory pathways (such as the PI3K/Akt and NF‐κB pathways) [22, 58]. Therefore, it remains unclear whether the neuroprotective effects of JB against CIRI are mediated solely through the JAK2/STAT3 pathway or involve synergistic interactions with other pathways, which warrants further investigation. Additionally, recovery and remodeling after brain injury is a long‐term and complex process [59]. This study mainly evaluated the efficacy of JB during the acute phase, and did not assess its sustained effects over longer time periods (such as 7, 14, or 28 days). Moreover, the estrous cycle in female animals can cause significant physiological and behavioral changes, which may interfere with experimental results. Using male rats helps to reduce variability and increase statistical power, thereby facilitating the detection of significant effects in preliminary mechanistic studies. In summary, future research should focus on exploring the multi‐pathway regulatory mechanisms of JB in CIRI, evaluating its long‐term efficacy, and identifying its direct molecular targets, so as to provide more comprehensive experimental evidence for JB as a potential therapeutic agent for stroke.

## 5 Conclusion

This study systematically elucidates that JB exerts protective effects in CIRI by inhibiting the JAK2/STAT3 signaling pathway, promoting the phenotypic transformation of microglia from the pro‐inflammatory M1 type to the anti‐inflammatory M2 type, thereby alleviating inflammation, enhancing neuroprotection, and facilitating tissue repair. These findings establish a pharmacological foundation for JB as an innovative candidate drug for IS treatment. However, several limitations remain in this study: First, the experimental data are derived solely from small animal models, and their therapeutic efficacy requires further validation in large animal models such as non‐human primates; second, mechanistic validation through preclinical models, such as human brain organoids, is lacking; additionally, the optimal therapeutic time window for JB remains unclear, and the long‐term safety of its administration requires systematic evaluation. Future studies should focus on translational research to further refine the pharmacological basis of JB.

## Author Contributions

**Yupeng Guo, Xuanwei Dong:** conceived and designed the research, conducted experiments, and analyzed data. Drafted and revised the manuscript critically for important intellectual content. **Min Liu, Dongsheng Liu, Jianxin Wang:** contributed to the acquisition, analysis, and interpretation of data. Provided substantial intellectual input during the drafting and revision of the manuscript. **Yupeng Guo, Shewei Guo:** participated in the conception and design of the study. Played a key role in data interpretation and manuscript preparation. All authors have read and approved the final version of the manuscript.

## Ethics Statement

This study was approved by The First Affiliated Hospital of Zhengzhou University Animal Ethics Committee (Approval No.: 2021090201).

## Consent

The authors have nothing to report.

## Conflicts of Interest

The authors declare no conflicts of interest.

## Data Availability

The data that support the findings of this study are available from the corresponding author, upon reasonable request.
